# QSPR modeling of physicochemical properties of SSRI and SNRI antidepressants using advanced molecular graph descriptors

**DOI:** 10.1038/s41598-026-65586-2

**Published:** 2026-08-07

**Authors:** Aqsa Kabeer, Zeeshan Saleem Mufti, Abdulrahman A. Almehizia, Ahmad A. Assiri, Gamachu Adugna Ganati

**Affiliations:** 1 Department of Mathematics and Statistics, The University of Lahore, Lahore, Pakistan; 2 Drug Exploration and Development Chair (DEDC), Department of Pharmaceutical Chemistry, College of Pharmacy, King Saud University, Riyadh, 11451 Saudi Arabia; 3 Department of Pharmacognosy, College of Pharmacy, Najran University, Najran, Saudi Arabia; 4 Department of Mathematics, Wallaga University, Nekemte, Ethiopia

**Keywords:** Chemistry, Drug discovery, Mathematics and computing

## Abstract

The Quantitative Structure–Property Relationship (QSPR) modeling is a successful computational technique that is capable of predicting physicochemical properties on the basis of the molecular structure. Twenty-five commonly used antidepressant drugs were used in the present study and a complete QSPR analysis was carried out by using neighborhood degree-based topological indices obtained from chemical graph theory. Molecular graphs, with the hydrogen atoms suppressed, were calculated nine topological descriptors; the correlations between these descriptors and eight measured physicochemical properties (boiling point, molar volume, molar refractivity, molecular weight, heavy atom count, molecular complexity, enthalpy of vaporization, and melting point) were computed. There were development and statistical assessment of three regression models (linear, quadratic and logarithmic) using coefficient of determination ($R^{2}$), F statistic, p-value, and standardized residuals. The comparative analysis showed that for the boiling point, the quadratic regression model is generally the most accurate predicting model, with a maximum $R^{2}$ value: 0.887 for the $ND_1(G)$ descriptor. Moreover, the two most informative neighborhood descriptors that consistently emerged as the most informative neighborhood descriptors for predicting several important physicochemical properties were $ND_1(G)$ and $ND_5(G)$. The developed regression equations are reliable and efficient predictive models, and do not require expensive experimental measurements during the early stages of drug discovery and require only molecular topology. The results of the present study indicate that the neighborhood degree-based topological indices are efficient tools for the development of QSPR models and can be applied in the rational design, screening and optimization of novel antidepressant drugs.

## Introduction

Depression and anxiety are among the mental health conditions that pose a major and increasingly serious social health issue in children, adolescents, and young adults. It is an issue significantly increased in the case of the significant segment of the population of youth who consistently have chronic pain. According to recent extensive evidence, it has turned out that about one out of every three young people with chronic pain fit the diagnostic criteria of an anxiety disorder, and one out of eight fit the diagnostic criteria of a depressive disorder^1,10^. This comorbidity is not just coincidental but is defined by a complicated, bidirectional relationship in which psychological suffering and physical pain interact to the extent of increased pain levels, increased functional impairment, and reduced quality of life is caused in most instances, by comorbidity^2^. This overlapping of chronic pain and mood disorders forms a very vulnerable group, and in whom the use of pharmacological intervention is a significant aspect of a multidisciplinary approach to treatment.

Empirical synthesis and experimental screening has been the basis of finding and optimizing new therapeutic agents over decades, which are often resource intensive, time consuming and costly. Counting on this, the discipline of computational chemistry has come up with effective predictive models that have simplified this task. Quantitative Structure-Property Relationship (QSPR) modeling has become one of the pillars among them. QSPR is based on the universal idea that the physicochemical and biological properties of a molecule are implicit functions of the geometry and electronic structure. Quantifying the molecular structure and defining it in numerical terms, and then associating the numbers with experimental results, QSPR can be used to predict properties of new, unsynthesized compounds.

In recent years, chemists have made remarkable efforts in the use of graph-theoretical descriptors for QSPR modelling, especially by improving the degree-based and distance-based topological indices. Recent studies (2023-2026) have demonstrated that these descriptors are able to accurately represent subtle structural changes in molecular graphs and have good predictive ability in regression-based approaches to physicochemical and pharmacological properties when used within these approaches^3^. Furthermore, contemporary studies focus on the incorporation of polynomial descriptors like M-polynomials and reduced reverse degree indices that enhance structural sensitivity and minimize degeneracy problems in conventional topological descriptors^3^. Moreover, hybrid methods that integrate classical graph descriptors with machine learning methods and nonlinear regression models have shown to be more accurate and generalizable in QSPR/QSAR prediction^4,5^. More recently, in drug discovery, it has been demonstrated that graph-theoretical approaches continue to be powerful tools for predicting biological activity and physicochemical properties, particularly in conjunction with sophisticated statistical and AI-driven modelling methods^6^. In general, the literature 2023–2026 is unanimous in emphasizing the relevance and development of graph-theoretical descriptors in QSPR studies of complex molecular systems.

One of the most abundant sources of molecular descriptors is the Chemical Graph Theory (CGT). In this formalism, a molecule is abstracted as a mathematical graph wherein atoms are represented as vertices and chemical bonds as edges. Topological indices are graph-theoretical invariants calculated from this molecular graph that encode information about size, shape, branching, symmetry, and cyclicity without requiring three-dimensional coordinates. These indices, such as the Zagreb indices, Randić connectivity index, and various degree-based descriptors, have proven exceptionally valuable in QSPR studies for predicting a wide array of properties, including boiling point, molar refractivity, toxicity, and biological activity. Their computational simplicity, minimal need for conformational analysis, and proven correlation with key properties make them ideal descriptors for high-throughput virtual screening. Despite the successful application of topological indices in modeling properties for various chemical classes, a focused and systematic QSPR investigation of modern antidepressant drugs specifically Selective Serotonin Reuptake Inhibitors (SSRIs) and Serotonin-Norepinephrine Reuptake Inhibitors (SNRIs) remains an underexplored area. These compounds, which include Fluoxetine, Sertraline, Escitalopram, and Venlafaxine, share core pharmacophores but exhibit distinct structural nuances that translate into varied pharmacokinetic and pharmacodynamic profiles. The medicinal chemists would be given a helpful instrument by having a strong, descriptive model that could predict their key physicochemical characteristics (e.g., melting point) with a high degree of accuracy. Such technology^7,8^can inform the rational development of the next generation antidepressants that have optimized bioavailability, selectivity, and safety profiles.

This paper will thus fill this gap with a thorough QSPR study of twenty-five popular antidepressant drugs in a set of nine topological indices. These are our three main goals: (1) to compute meaningful topological descriptors of each of the antidepressant compounds and compile their experimental physicochemical properties; (2) to build, test, compare performance of linear, quadratic and logarithmic regression models of each pair of property-descriptors; and (3) to determine which topological index best predicts each significant property and to write the robust regression equation. This research focuses on the construction of a computational framework for early prediction and optimization of antidepressant drugs based on graph theory and its ability to correlate molecular topology and physicochemical properties, which would be helpful in rational drug design and improve the time efficiency of development of new antidepressant drugs^9,10^. This study seeks to create a method using graph theory that can be used in the initial screening and optimization of antidepressant compounds to get a better understanding of the structure–property relationships that could be used in the future to investigate therapies for chronic pain and mood disorders^11,12^.

**Fluoxetine** – Fluoxetine belongs to a group of medications known as selective serotonin reuptake inhibitors (SSRIs), which boost levels of serotonin in the brain and is used to treat major depression and anxiety disorders^17^.

**Sertraline** – Sertraline is an SSRI with a good efficacy and safety profile as a first line intervention for depression and anxiety^15^.

**Escitalopram** – Escitalopram is a highly selective SSRI which has been shown to be well-tolerated and effective in major depressive disorder^17^.

**Paroxetine** – Paroxetine is an SSRI proven to be effective in depression and anxiety disorders and is sometimes limited by withdrawal effects and side effects^14,15^.

**Citalopram** – Citalopram is a SSRI that increases serotonergic neurotransmission, and is still effective for treating depressive disorders^16^.

**Venlafaxine** – Venlafaxine is a medication belonging to a class of drugs called serotonin-norepinephrine reuptake inhibitors (SNRIs) which boost serotonin and norepinephrine in the brain, thereby helping to alleviate depressive symptoms^15^.

**Duloxetine** – Duloxetine is a serotonergic noradrenergic reuptake inhibitor (SNRI) approved for depression and chronic pain medications^16^.

**Desvenlafaxine** is the active metabolite of venlafaxine, which is an SNRI that helps to reduce the symptoms of major depressive disorder (MDD)^17^.

The antidepressant and anxiolytic properties of fluvoxamine make it particularly beneficial for people who experience obsessive-compulsive symptoms^16^.

**Vilazodone** – Vilazodone is a multimodal antidepressant, which has serotonin reuptake inhibition and partial agonism at 5-HT1A receptors^15^.

**Bupropion** – Bupropion is a norepinephrine-dopamine reuptake inhibitor (NDRI) with relatively few sexual side effects and by improving the depression symptoms^16^.

**Mirtazapine** – Mirtazapine is a noradrenergic and specific serotonergic antidepressant (NaSSA) that is useful especially in depressed patients with insomnia and weight loss^16^.

**Trazodone** – Trazodone is a serotonin antagonist and reuptake inhibitor (SARI) that is often prescribed for depression with insomnia due to its sedative effects^17^.

**Nefazodone** – Nefazodone is a serotonin receptor antagonist and serotonin reuptake inhibitor (SARI), which has antidepressant properties^15^.

**Vortioxetine** – Vortioxetine is a multimodal antidepressant that acts on various serotonin receptors and enhances mood and cognitive dysfunction associated with depression^15^.

**Nortriptyline** – Nortriptyline is a tricyclic antidepressant (TCA) which is effective in treatment-resistant depression^16^ and inhibits norepinephrine reuptake.

**Imipramine** – Imipramine is a TCA that blocks the reuptake of serotonin and norepinephrine, and is a well-established treatment for major depressive disorder^17^.

**Clomipramine** is a TCA with high serotonergic activity, and has been shown to be effective in the treatment of depressive and obsessive-compulsive disorders^13^.

**Desipramine** – Desipramine is a TCA which acts primarily as a norepinephrine reuptake inhibitor and is effective at treating depressive symptoms^16^.

**Phenelzine** – Phenelzine is an irreversible MAOI that is effective in refractory and atypical depression^13^.

**Isocarboxazid** – Isocarboxazid is an MAOI with an increasing concentration of monoamines and is still effective in treatment-resistant depressive disorders^13^.

**Amoxapine** – Amoxapine is a tetracyclic antidepressant that has antidepressant and antipsychotic properties because of its ability to affect monoaminergic pathways^17^.

**Mianserin** – Mianserin is a tetracyclic antidepressant that has been found to increase noradrenergic and serotonergic transmission by *α*2-adrenergic antagonism^16^.

**Reboxetine** is a selective norepinephrine reuptake inhibitor (NRI) which has an antidepressant effect through noradrenergic neurotransmission^15^.

**Tianeptine** – Tianeptine is an atypical antidepressant that alter the activity of *μ*-opioid receptors and the glutamatergic neurotransmission to exert antidepressant activity^17^.

## Preliminaries

See Table 1.

**Table 1:** Preliminary Definitions and Notations.

Definition	Term	Description	Mathematical Expression
Definition 1	Graph	Graph theory provides the concept of a graph through which we can demonstrate the relationship between objects. It consists of two sets: a vertex set and an edge set, respectively.	*G=(V(G),E(G))*
Definition 2	Order of a Graph	The order of the graph indicates the number of vertices in the graph. This is represented by *O*(*G*).	*O(G)=|V(G)|*
Definition 3	Size of a Graph	The size of the graph indicates the number of edges in the graph. This is represented by |*E*(*G*)|.	|*E*(*G*)|
Definition 4	Neighborhood of a Vertex	Let *G=(V(G),E(G))* be a graph, where *V*(*G*) and *E*(*G*) denote the vertex set and edge set of *G*, respectively. For a vertex *u ∈ V(G)*, the neighborhood of *u*, denoted by *N*(*u*), is the set of all vertices adjacent to *u*. Mathematically,	$\displaystyle N(u)=\{\,v\in V(G)\mid uv\in E(G)\,\}$
Definition 5	First and Second Zagreb Indices	The first and second Zagreb indices are considered to be the fundamental indices in chemical graph theory. This index is present in a degree based version, but here we consider the neighborhood version, which can be explained mathematically as follows^18^:	$\displaystyle NM_1(G)=\sum _{v\in V(G)}\delta (v)^2$ $\displaystyle NM_2(G)=\sum _{uv\in E(G)}\delta (u)\delta (v)$
Definition 6	Neighborhood Atom Bond Connectivity Index	The main focus of the neighborhood is to explain the atomic connectivity, bond strength, and number of bonds with a single atom. Therefore, each index explains the connectivity of atoms differently when the neighborhood attachment is explained. Similarly, the neighborhood atom bond connectivity performs the same task and is explained as follows^19^:	$\displaystyle NABC(G)=\sum _{uv\in E(G)} \sqrt{\frac{\delta (u)+\delta (v)-2}{\delta (u)\delta (v)}}$
Definition 7	Neighborhood Forgotten Index	The Neighbourhood Forgotten Index is equal to the sum of the cubes of the neighbourhood degree of each vertex of a graph	$\displaystyle NF^{*}(G)=\sum _{v\in V(G)}\delta (v)^3$
Definition 8	First NDe Index	Neighborhood degree product ($ND_1(G)$) belongs to the family of NDe indices, which influence our results related to different properties of drugs, and our first index of this family is the neighborhood version of the reciprocal Randic index^19^.	$\displaystyle ND_1(G)= \sum _{uv\in E(G)}\sqrt{\delta (u)\delta (v)}$
Definition 9	Second NDe Index	The second NDe index ($ND_2(G)$) is the sum connectivity index in the neighborhood, which is defined as follows^19^:	$\displaystyle ND_2(G)= \sum _{uv\in E(G)} \frac{1}{\sqrt{\delta (u)+\delta (v)}}$
Definition 10	Third NDe Index	The third NDe ($ND_3(G)$), also called the mixed term index, is written in the following order^19^:	$\displaystyle ND_3(G)= \sum _{uv\in E(G)} \delta (u)\delta (v)[\delta (u)+\delta (v)]$
Definition 11	Fourth NDe Index	We can define the fourth NDe index ($ND_4(G)$) just like the Randic index as^19^:	$\displaystyle ND_4(G)= \sum _{uv\in E(G)} \frac{1}{\sqrt{\delta (u)\delta (v)}}$
Definition 12	Fifth NDe Index	The fifth member of NDe ($ND_5(G)$) is useful because of its predictive power for different properties, and we calculate it as:	$\displaystyle ND_5(G)= \sum _{uv\in E(G)} \left[ \frac{\delta (u)}{\delta (v)}+ \frac{\delta (v)}{\delta (u)}\right]$

## Methodology and analysis

**Figure:**
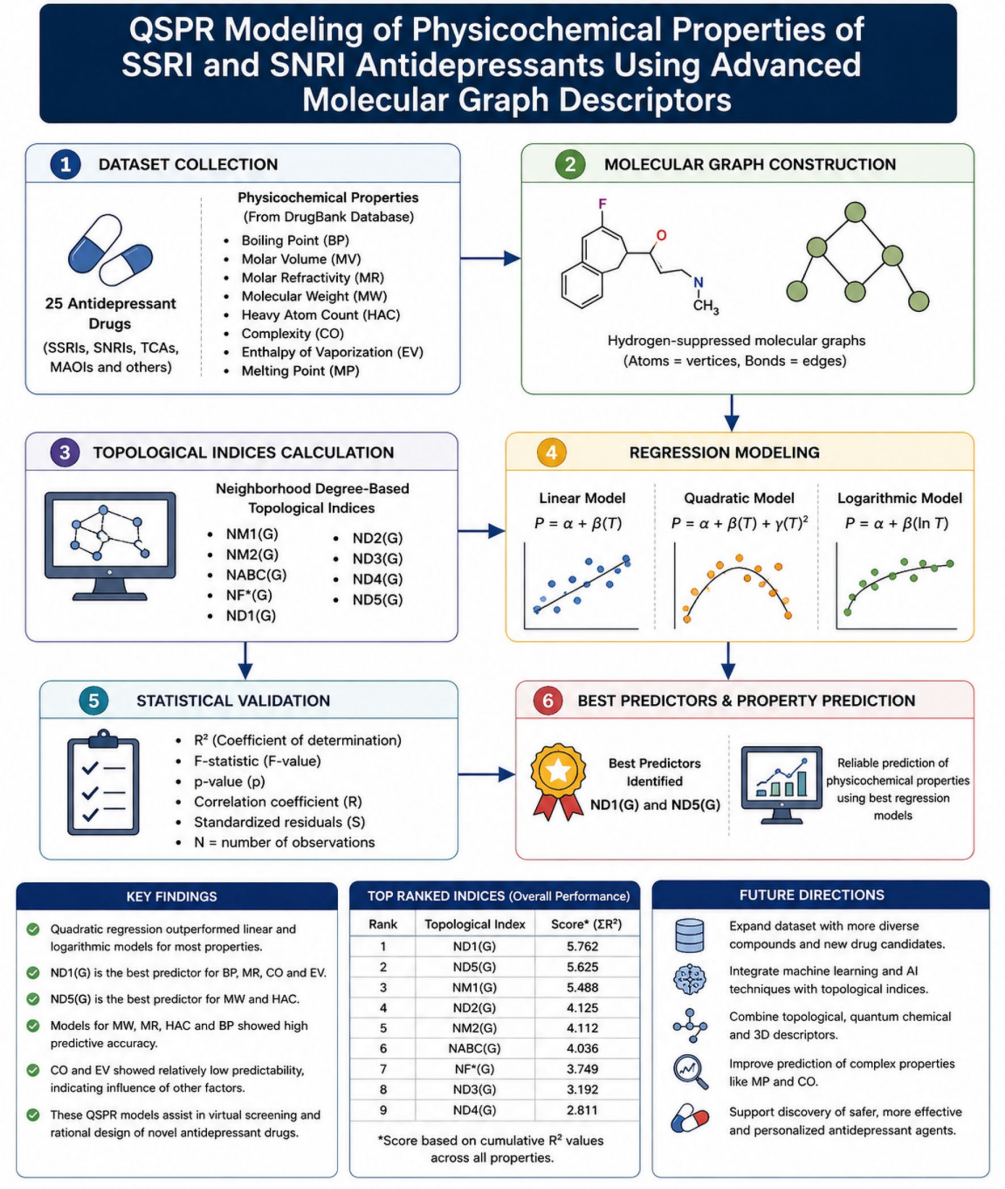

Here, to model eight physical properties of twenty-five drugs, Fluoxetine, Sertraline, Escitalopram, Paroxetine, Citalopram, Venlafaxine, Duloxetine, Desvenlafaxine, Fluvoxamine, Vilazodone, Bupropion, Mirtazapine, Trazodone, Nefazodone, Vortioxetine, Nortriptyline, Imipramine, Clomipramine, Desipramine, Phenelzine, Isocarboxazid, Amoxapine, Mianserin, Reboxetine and Tianeptine, nine topological indices are used. The indices would be used to calculate the respective numerical values that are related to each physical property.

**Fig. 1:**
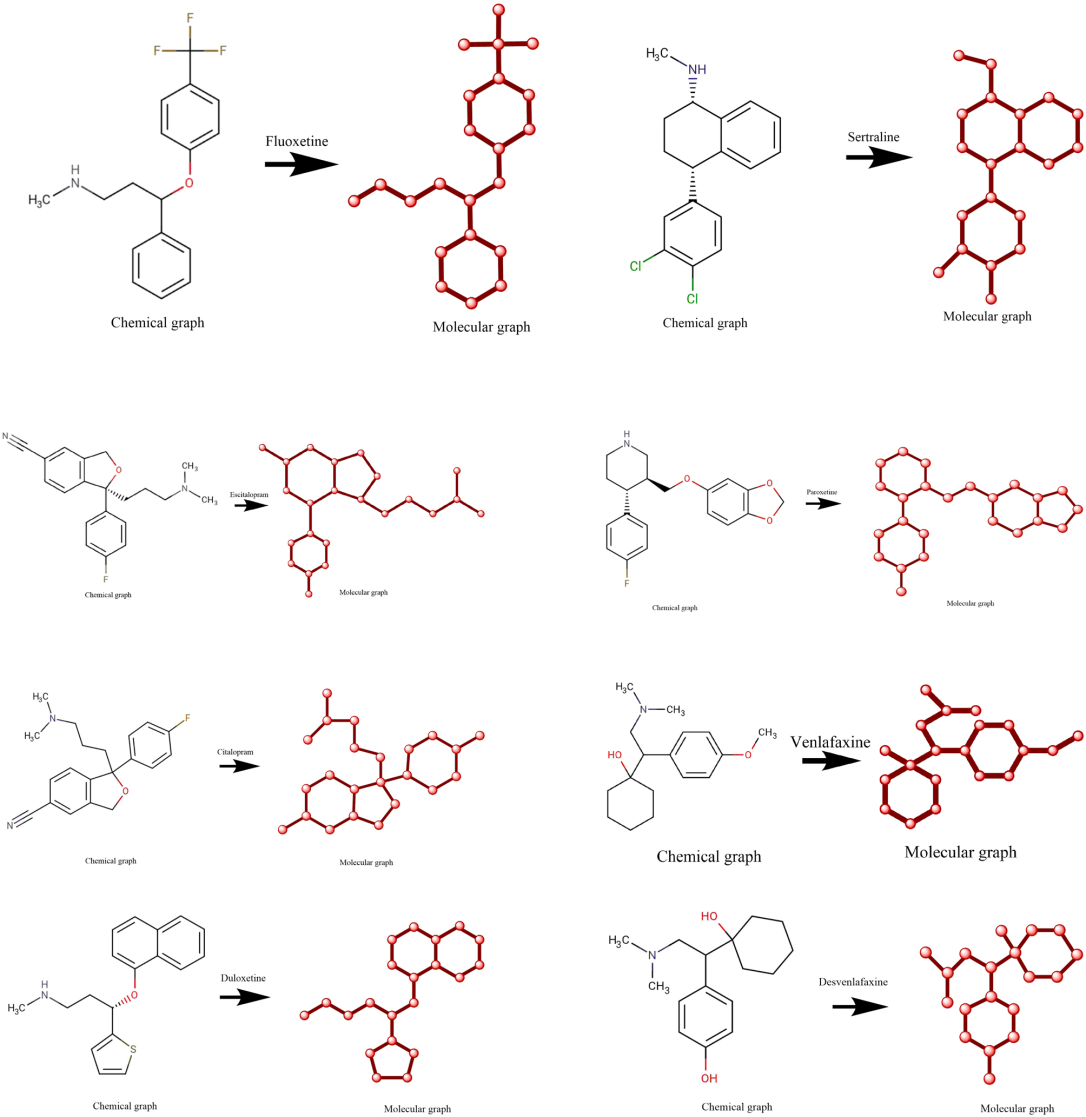
Chemical and molecular structures of the drugs.

The chemical structures of the selected drugs were transformed into molecular graphs by representing each non-hydrogen atom as a vertex and each covalent bond as an edge. The connectivity and branching patterns of the molecules were preserved during this conversion, resulting in hydrogen-suppressed molecular graphs. These graph representations were then used to compute topological descriptors and perform QSPR analysis of the drug properties. Figure 1 shows the chemical and molecular structures of first eight drugs. Click here to view remaining molecular graphs

The structural relationship between the eight biochemical compounds was analyzed using the edge distribution analysis. The distribution of the edge of each of the compounds is presented in the following Table 2 to make it easier to comprehend. The present analysis contributes to a better insight into the structural characteristics and networks of interaction of these compounds, thus leading to the better understanding of their chemical structure and possible functions in biology.

**Table 2:** Neighborhood degree-based edge partitions of the selected antidepressant drugs.

Drug	Edge Partition (degree pair : count)
Fluoxetine	$(2,3)\!:\!1,\ (3,4)\!:\!1,\ (4,4)\!:\!2,\ (4,6)\!:\!3,\ (5,4)\!:\!3,\ (5,5)\!:\!2,\ (5,6)\!:\!2,\ (5,7)\!:\!3,\ (5,8)\!:\!2,\ (6,6)\!:\!1,\ (6,7)\!:\!1,\ (6,8)\!:\!1,\ (7,7)\!:\!1$
Sertraline	$(2,4)\!:\!1,\ (4,4)\!:\!1,\ (4,7)\!:\!1,\ (5,4)\!:\!2,\ (5,5)\!:\!2,\ (5,6)\!:\!1,\ (5,7)\!:\!2,\ (5,8)\!:\!3,\ (6,3)\!:\!2,\ (6,6)\!:\!1,\ (6,7)\!:\!2,\ (8,7)\!:\!2,\ (8,8)\!:\!2$
Escitalopram	$(3,4)\!:\!2,\ (3,5)\!:\!2,\ (5,4)\!:\!3,\ (5,5)\!:\!5,\ (5,6)\!:\!2,\ (5,7)\!:\!5,\ (6,7)\!:\!1,\ (6,8)\!:\!1,\ (7,8)\!:\!1,\ (7,9)\!:\!2,\ (9,8)\!:\!1$
Paroxetine	$(4,4)\!:\!1,\ (4,5)\!:\!4,\ (5,3)\!:\!1,\ (5,5)\!:\!6,\ (5,6)\!:\!2,\ (5,7)\!:\!7,\ (5,8)\!:\!1,\ (6,6)\!:\!1,\ (6,7)\!:\!1,\ (7,7)\!:\!1,\ (7,8)\!:\!2$
Citalopram	$(2,4)\!:\!1,\ (3,4)\!:\!2,\ (4,4)\!:\!2,\ (4,6)\!:\!4,\ (4,8)\!:\!1,\ (5,5)\!:\!2,\ (5,6)\!:\!2,\ (5,7)\!:\!2,\ (6,8)\!:\!1,\ (6,9)\!:\!1,\ (9,7)\!:\!1,\ (9,8)\!:\!1$
Venlafaxine	$(2,4)\!:\!1,\ (3,4)\!:\!2,\ (4,4)\!:\!2,\ (4,6)\!:\!4,\ (4,8)\!:\!1,\ (5,5)\!:\!2,\ (5,6)\!:\!2,\ (5,7)\!:\!2,\ (6,8)\!:\!2,\ (6,9)\!:\!1,\ (9,7)\!:\!1,\ (9,8)\!:\!1$
Duloxetine	$(2,3)\!:\!1,\ (3,4)\!:\!1,\ (4,4)\!:\!2,\ (4,5)\!:\!7,\ (5,7)\!:\!6,\ (5,8)\!:\!1,\ (6,7)\!:\!2,\ (7,7)\!:\!1,\ (7,8)\!:\!2$
Desvenlafaxine	$(3,4)\!:\!3,\ (4,4)\!:\!2,\ (4,6)\!:\!3,\ (4,8)\!:\!1,\ (5,4)\!:\!2,\ (5,5)\!:\!2,\ (6,8)\!:\!2,\ (6,9)\!:\!1,\ (7,5)\!:\!2,\ (7,9)\!:\!1,\ (8,9)\!:\!1$
Fluvoxamine	$(2,3)\!:\!2,\ (3,4)\!:\!2,\ (4,4)\!:\!3,\ (4,5)\!:\!2,\ (4,6)\!:\!3,\ (5,5)\!:\!2,\ (5,7)\!:\!2,\ (5,8)\!:\!4,\ (6,8)\!:\!1,\ (7,8)\!:\!1$
Vilazodone	$(3,5)\!:\!3,\ (4,4)\!:\!1,\ (4,5)\!:\!2,\ (5,5)\!:\!6,\ (5,6)\!:\!6,\ (5,7)\!:\!10,\ (6,7)\!:\!4,\ (6,8)\!:\!1,\ (7,7)\!:\!1,\ (7,8)\!:\!2$
Bupropion	$(3,5)\!:\!1,\ (3,6)\!:\!1,\ (3,7)\!:\!1,\ (4,5)\!:\!5,\ (5,5)\!:\!1,\ (5,6)\!:\!1,\ (5,7)\!:\!2,\ (6,7)\!:\!3,\ (7,7)\!:\!1$
Mirtazapine	$(3,5)\!:\!1,\ (4,4)\!:\!2,\ (4,5)\!:\!4,\ (5,5)\!:\!2,\ (5,6)\!:\!1,\ (5,7)\!:\!2,\ (5,8)\!:\!3,\ (6,7)\!:\!2,\ (6,8)\!:\!1,\ (7,8)\!:\!2,\ (8,8)\!:\!3$
Trazodone	$(3,5)\!:\!1,\ (3,7)\!:\!1,\ (4,4)\!:\!1,\ (4,5)\!:\!6,\ (5,5)\!:\!3,\ (5,6)\!:\!4,\ (5,7)\!:\!5,\ (5,8)\!:\!1,\ (6,7)\!:\!3,\ (7,7)\!:\!2,\ (7,8)\!:\!2$
Nefazodone	$(2,3)\!:\!1,\ (3,4)\!:\!1,\ (3,5)\!:\!1,\ (3,7)\!:\!1,\ (4,4)\!:\!2,\ (4,5)\!:\!9,\ (4,7)\!:\!1,\ (5,5)\!:\!3,\ (5,6)\!:\!7,\ (5,7)\!:\!6,\ (6,7)\!:\!1,\ (7,7)\!:\!3$
Vortioxetine	$(3,5)\!:\!1,\ (3,6)\!:\!1,\ (4,4)\!:\!3,\ (4,5)\!:\!4,\ (5,5)\!:\!2,\ (5,6)\!:\!1,\ (5,7)\!:\!4,\ (5,8)\!:\!1,\ (6,6)\!:\!1,\ (6,7)\!:\!3,\ (7,8)\!:\!2$
Nortriptyline	$(2,3)\!:\!1,\ (3,4)\!:\!1,\ (4,4)\!:\!3,\ (4,5)\!:\!5,\ (5,5)\!:\!1,\ (5,7)\!:\!4,\ (5,8)\!:\!3,\ (7,8)\!:\!2,\ (8,8)\!:\!2$
Imipramine	$(3,4)\!:\!2,\ (4,4)\!:\!2,\ (4,5)\!:\!7,\ (5,5)\!:\!1,\ (5,7)\!:\!4,\ (5,8)\!:\!3,\ (7,8)\!:\!2,\ (8,8)\!:\!2$
Clomipramine	$(3,4)\!:\!2,\ (3,5)\!:\!1,\ (4,4)\!:\!1,\ (4,5)\!:\!5,\ (5,5)\!:\!3,\ (5,6)\!:\!1,\ (5,7)\!:\!4,\ (5,8)\!:\!2,\ (6,8)\!:\!1,\ (7,8)\!:\!2,\ (8,8)\!:\!2$
Desipramine	$(2,3)\!:\!1,\ (3,4)\!:\!1,\ (4,4)\!:\!3,\ (4,5)\!:\!5,\ (5,5)\!:\!2,\ (5,7)\!:\!5,\ (5,8)\!:\!2,\ (7,8)\!:\!1$
Phenelzine	$(2,3)\!:\!1,\ (3,4)\!:\!1,\ (4,4)\!:\!2,\ (4,5)\!:\!3,\ (5,6)\!:\!3$
Isocarboxazid	$(3,5)\!:\!1,\ (3,6)\!:\!1,\ (4,4)\!:\!2,\ (4,5)\!:\!4,\ (5,5)\!:\!2,\ (5,6)\!:\!5,\ (5,7)\!:\!1,\ (6,7)\!:\!2$
Amoxapine	$(3,5)\!:\!1,\ (4,4)\!:\!3,\ (4,5)\!:\!4,\ (5,5)\!:\!2,\ (5,6)\!:\!1,\ (5,7)\!:\!5,\ (6,7)\!:\!3,\ (6,8)\!:\!2,\ (7,7)\!:\!1,\ (7,8)\!:\!2,\ (8,8)\!:\!1$
Mianserin	$(3,5)\!:\!1,\ (4,4)\!:\!2,\ (4,5)\!:\!4,\ (5,5)\!:\!2,\ (5,6)\!:\!1,\ (5,7)\!:\!2,\ (5,8)\!:\!3,\ (6,7)\!:\!2,\ (6,8)\!:\!1,\ (7,8)\!:\!2,\ (8,8)\!:\!3$
Reboxetine	$(2,3)\!:\!1,\ (3,5)\!:\!1,\ (4,4)\!:\!5,\ (4,5)\!:\!4,\ (4,6)\!:\!2,\ (4,8)\!:\!1,\ (5,7)\!:\!4,\ (5,8)\!:\!1,\ (6,8)\!:\!3,\ (6,9)\!:\!1,\ (7,8)\!:\!1,\ (7,9)\!:\!1,\ (8,9)\!:\!1$
Tianeptine	$(3,4)\!:\!2,\ (3,5)\!:\!1,\ (3,8)\!:\!1,\ (4,4)\!:\!5,\ (4,5)\!:\!5,\ (4,8)\!:\!2,\ (5,5)\!:\!2,\ (5,6)\!:\!1,\ (5,8)\!:\!4,\ (6,9)\!:\!1,\ (8,8)\!:\!5,\ (8,9)\!:\!2$

### Physicochemical characteristics of drugs

Table 3 provides an overview of the physical and chemical characteristics of the drugs under study such as boiling point, molar volume, molar refractivity, molecular weight, number of heavy atoms, complexity, enthalpy of vaporization, and melting point. These drugs have topological descriptors that are shown in Table 4. The SPSS was used to perform regression analysis. The DrugBank database provided all the information related to drugs (https://go.drugbank.com).

**Table 3:** Physicochemical Properties of the Selected Antidepressant Drugs.

Sr.No	Drugs	${BP} (^0 C)$	${MV} ({cm}^3/)$	${MR} ({cm}^3/)$	${MW} ({g/mol})$	HAC	CO	EV (${KJ/mol}$)	${MP} (^0 C)$
1	Fluoxetine	395	266.7	79.9	309.33	22	296	64.5	179
2	Sertraline	416.3	243.9	85.8	306.23	20	315	67	246
3	Escitalopram	428.3	272.6	92.1	324.392	24	401	68.3	146
4	Paroxetine	451.7	271.5	87.9	329.371	24	396	71.1	120
5	Citalopram	428.3	272.6	92.1	324.392	24	403	68.3	156
6	Venlafaxine	397.6	261.7	82.6	277.40	20	285	68.3	215
7	Duloxetine	466.2	256.8	91.1	297.4146	21	373	72.8	176
8	Desvenlafaxine	403.8	236.1	77.8	263.381	19	283	69.1	232
9	Fluvoxamine	370.6	27.3	76.8	434.4	30	446	61.7	120
10	Vilazodone	745.1	328.8	128.7	441.5	33	729	108.6	227
11	Bupropion	334.8	224.7	67.9	239.74	16	247	57.8	233
12	Mirtazapine	432.4	216.6	80.7	265.35	20	345	68.8	114
13	Trazodone	528.5	278.8	104.2	371.9	26	611	80.3	86
14	Nefazodone	599.6	380.7	133.1	470	33	649	89.3	83
15	Vortioxetine	424.8	256.5	92.7	298.4	21	316	67.9	115
16	Nortriptyline	403.4	242.8	86.8	263.4	20	307	65.5	213
17	Imipramine	403.1	269.2	88.9	280.4	21	291	65.4	174
18	Clomipramine	434.2	281.2	93.8	314.9	22	346	69.0	189
19	Desipramine	407.4	254.3	84.2	266.4	20	267	65.9	214
20	Phenelzine	281.4	134.7	42.9	136.19	10	77.3	52.0	157
21	Isocarboxazid	394.5	191.6	63.2	231.25	17	254	64.5	105
22	Amoxapine	469.9	228.2	86.8	313.8	22	424	73.2	175
23	Mianserin	411.3	223.7	82.9	264.4	20	342	66.4	87
24	Reboxetine	443.7	281.5	90.1	313.4	23	333	70.1	170
25	Tianeptine	609.2	316.5	113.9	437	29	654	95.1	148

**Table 4:** Selected Antidepressant Drugs and their computed topological values.

Sr.No	Drugs	$NM_{1}(G)$	*NABC*(*G*)	$NM_{2}(G)$	$NF^*(G)$	$ND_{1}(G)$	$ND_{2}(G)$	$ND_3(G)$	$ND_4(G)$	$ND_5(G)$
1	Fluoxetine	242	12.9971	652	1358	119.7877	7.2377	7426	4.6767	47.8667
2	Sertraline	255	12.0878	758	1589	125.7606	6.5998	9650	4.0987	46.9417
3	Escitalopram	280	13.8200	818	1686	138.8775	7.6359	10256	4.7770	51.7540
4	Paroxetine	300	14.8642	845	1740	148.9005	8.1886	9932	5.0402	55.6179
5	Citalopram	285	13.9152	858	1823	140.2641	7.6423	11552	4.8527	53.4683
6	Venlafaxine	230	11.8056	657	1384	113.4234	6.5230	8202	4.1960	44.5393
7	Duloxetine	246	12.9322	685	1416	121.9677	7.2134	8128	4.6532	47.5940
8	Desvenlafaxine	217	11.2544	617	1297	107.1499	6.2401	7698	4.0090	41.9893
9	Fluvoxamine	271	19.8085	640	1357	133.5052	11.6432	6924	8.9344	67.9964
10	Vilazodone	406	19.7242	1158	2386	201.4052	10.8344	13750	6.6292	74.4571
11	Bupropion	170	9.0439	454	954	83.8105	4.9642	5136	3.1566	34.1119
12	Mirtazapine	272	12.4353	833	1718	134.9009	6.8257	10842	4.1428	47.5702
13	Trazodone	324	15.9853	918	1900	160.5111	8.7818	10918	5.4175	60.3655
14	Nefazodone	372	20.4531	978	2028	184.2277	11.3740	10812	7.3499	74.9929
15	Vortioxetine	250	12.8272	695	1438	123.8791	7.0840	8166	4.4520	47.7893
16	Nortriptyline	290	19.3949	751	1564	143.3738	11.3440	8916	8.5152	67.3345
17	Imipramine	252	12.8330	721	1496	124.8378	7.1157	8902	4.5103	47.6845
18	Clomipramine	266	13.3246	768	1588	131.8474	7.3730	9516	4.6401	49.7429
19	Desipramine	228	12.5312	607	1278	112.6362	6.9959	6918	4.5762	45.9893
20	Phenelzine	88	5.9953	200	408	43.7617	3.4368	1900	2.4155	20.5000
21	Isocarboxazid	182	10.2753	464	956	90.2679	5.7107	4920	3.6788	37.2952
22	Amoxapine	286	13.6492	845	1732	142.0844	7.5251	10526	4.6171	51.3452
23	Mianserin	272	12.4353	833	1718	134.9009	6.8257	10842	4.1428	47.5702
24	Reboxetine	287	14.5444	830	1745	141.6936	8.0658	10472	5.1767	54.6607
25	Tianeptine	354	17.1925	1065	2246	174.4690	9.4594	14148	5.9731	65.8528

Topological indices like first and second Zagreb indices, neighborhood atom bond connectivity, Neighbourhood forgotten index, Neighborhood degree products are extensively used in the analysis of QSPR since they have a quantitative representation of molecular structure by reflecting the characteristics that concern connectivity, branching, and global complexity. These descriptors have been found useful in the estimation of different physicochemical and biological properties such as, boiling point, solubility, stability and biological activity. The neighborhood-based topological indices $NM_{1}(G)$, $NM_{2}(G)$, *NABC*(*G*), $NF^{*}(G)$ and $ND_{1}(G)$ to $ND_{5}(G)$ are numerical descriptors that are derived from a molecular graph (G), in which vertices correspond to atoms and edges to chemical bonds. These indices, which include neighborhood degree information, describe the local connectivity, branching patterns, and structural complexity, of a molecule. Specifically, $NM_{1}(G)$ and $NM_{2}(G)$ give information on the interaction between adjacent atomic environments, *NABC*(*G*) gives information on the molecular irregularity and bond heterogeneity, $NF^{*}(G)$ gives information on the distribution of neighboring atomic contributions, and the indices $ND_{1}(G)$ to $ND_{5}(G)$ give information on neighboring degree interactions and the molecular branching.

For ease of comprehension and better understanding, the complete computation of the second neighborhood Zagreb index is carried out step-by-step by calculating first the ordinary degrees of all vertices in a graph and then calculating the neighborhood degrees of all the vertices. There are 21 vertices, with 1 vertex of degree 4, 4 vertices of degree 1, 5 vertices of degree 3, and 11 vertices of degree 2. Using the definition

$$\delta (v)=\sum _{u\in N(v)} d(u),$$

the neighborhood degrees of all vertices are obtained as

$$\{6,4,4,4,8,5,5,5,5,7,8,5,4,3,2,7,5,5,4,4,4\}.$$

The second neighborhood index of Zagreb is then the sum of the degree product of neighborhoods of all the edges,

$$NM_{2}(G)=\sum _{uv\in E(G)} \delta (u)\delta (v),$$

and substituting the values of all 22 edges gives

$$NM_{2}(G)=652.$$

Hence, for the given graph, the second neighborhood Zagreb index is

$$\boxed {NM_{2}(G)=652},$$

providing a complete and systematic evaluation of this topological descriptor.

From the chemical point of view these descriptors are very similar to molecular size, compactness, branching and electronic distribution which significantly affect the physicochemical and pharmacological properties of drug molecules. The neighborhood-based indices are well suited to capture the structural information useful for QSPR modelling since they are strongly dependent on molecular topology. Hence, these indices are useful tools to establish statistical relationships between molecular structure and the properties of drugs, such as reliable estimation of some physicochemical properties and drug design and discovery. They, therefore, contribute greatly to providing credible structure-property relationships, as well as aiding in systematic development and evaluation of drugs. To provide a more clear and comparative results, the results of various regression methods are shown separately: Table 5 represents the outcomes of the linear regression model, Table 6 describes the results of quadratic regression analysis, and Table 7 synthesizes the results of the logarithmic regression model.

**Table 5:** Outcomes of Linear Regression Model.

Property	Predictors	$R^{2}$	Best Predictor
BP	$NM_{1}(G)$	0.771	$ND_1(G)$
	$NM_{2}(G)$	0.471	
	*NABC*(*G*)	0.743	
	$NF^{*}(G)$	0.734	
	$ND_1(G)$	0.773	
	$ND_2(G)$	0.397	
	$ND_3(G)$	0.626	
	$ND_4(G)$	0.198	
	$ND_5(G)$	0.573	
MV	$NM_{1}(G)$	0.347	No best predictor
	$NM_{2}(G)$	0.085	
	*NABC*(*G*)	0.407	
	$NF^{*}(G)$	0.403	
	$ND_1(G)$	0.349	
	$ND_2(G)$	0.050	
	$ND_3(G)$	0.383	
	$ND_4(G)$	0.001	
	$ND_5(G)$	0.147	
MR	$NM_{1}(G)$	0.807	$ND_1(G)$
	$NM_{2}(G)$	0.605	
	*NABC*(*G*)	0.810	
	${NF^{*}(G)}$	0.809	
	$ND_1(G)$	0.867	
	$ND_2(G)$	0.527	
	$ND_3(G)$	0.685	
	$ND_4(G)$	0.303	
	$ND_5(G)$	0.711	
MW	$NM_{1}(G)$	0.523	$ND_5(G)$
	$NM_{2}(G)$	0.580	
	*NABC*(*G*)	0.392	
	$NF^{*}(G)$	0.405	
	$ND_1(G)$	0.519	
	$ND_2(G)$	0.547	
	$ND_3(G)$	0.284	
	$ND_4(G)$	0.433	
	$ND_5(G)$	0.609	
HAC	$NM_{1}(G)$	0.830	$ND_5(G)$
	$NM_{2}(G$	0.803	
	*NABC*(*G*)	0.685	
	$NF^{*}(G)$	0.693	
	$ND_1(G)$	0.827	
	$ND_2(G)$	0.749	
	$ND_3(G)$	0.539	
	$ND_4(G)$	0.556	
	$ND_5(G)$	0.860	
CO	$NM_{1}(G$	0.714	$ND_1(G)$
	$NM_{2}(G)$	0.587	
	*NABC*(*G*)	0.625	
	$NF^{*}(G)$	0.621	
	$ND_1(G)$	0.715	
	$ND_2(G)$	0.530	
	$ND_3(G)$	0.504	
	$ND_4(G)$	0.346	
	$ND_5(G)$	0.649	
EV	$NM_{1}(G)$	0.725	$ND_1(G)$
	$NM_{2}(G)$	0.437	
	*NABC*(*G*)	0.706	
	$NF^{*}(G)$	0.701	
	$ND_1(G)$	0.727	
	$ND_2(G)$	0.367	
	$ND_3(G)$	0.605	
	$ND_4(G)$	0.181	
	$ND_5(G)$	0.536	
MP	$NM_{1}(G)$	0.040	No best predictor
	$NM_{2}(G)$	0.037	
	*NABC*(*G*)	0.029	
	$NF^{*}(G$	0.025	
	$ND_1(G)$	0.041	
	$ND_2(G)$	0.034	
	$ND_3(G)$	0.017	
	$ND_4(G)$	0.020	
	$ND_5(G)$	0.036

**Table 6:** Outcomes of Quadratic Regression Model.

Property	Predictors	$R^{2}$	Best Predictor
BP	$NM_{1}(G)$	0.885	$ND_1(G)$
	$NM_{2}(G)$	0.476	
	*NABC*(*G*)	0.869	
	$NF^{*}(G)$	0.858	
	$ND_1(G)$	0.887	
	$ND_2(G)$	0.444	
	$ND_3(G)$	0.700	
	$ND_4(G)$	0.561	
	$ND_5(G)$	0.586	
MV	$NM_{1}(G)$	0.353	No best predictor
	$NM_2(G)$	0.187	
	*NABC*(*G*)	0.410	
	$NF^{*}(G)$	0.406	
	$ND_1(G)$	0.355	
	$ND_2(G)$	0.257	
	$ND_3(G)$	0.383	
	$ND_4(G)$	0.586	
	$ND_5(G)$	0.170	
MR	$NM_{1}(G)$	0.877	$ND_1(G)$
	$NM_{2}(G)$	0.645	
	*NABC*(*G*)	0.816	
	$NF^{*}(G)$	0.816	
	$ND_1(G)$	0.878	
	$ND_2(G)$	0.632	
	$ND_3(G)$	0.685	
	$ND_4(G)$	0.751	
	$ND_5(G)$	0.713	
MW	$NM_1(G)$	0.542	No best predictor
	$NM_2(G)$	0.580	
	*NABC*(*G*)	0.406	
	$NF^{*}(G)$	0.421	
	$ND_1(G)$	0.537	
	$ND_2(G)$	0.551	
	$ND_3(G)$	0.288	
	$ND_4(G)$	0.543	
	$ND_5(G)$	0.619	
HAC	$NM_{1}(G)$	0.836	$ND_5(G)$
	$NNM_{2}(G)$	0.813	
	*NNABC*(*G*)	0.6889	
	$NNF^{*}(G)$	0.697	
	$NND_1(G)$	0.832	
	$ND_2(G)$	0.786	
	$ND_3(G)$	0.539	
	$ND_4(G)$	0.784	
	$NND_5(G)$	0.860	
CO	$NM_{1}(G)$	0.773	$ND_1(G)$
	$NM_{2}(G)$	0.588	
	*NABC*(*G*)	0.694	
	$NF^{*}(G)$	0.690	
	$ND_1(G)$	0.774	
	$ND_2(G)$	0.553	
	$ND_3(G)$	0.548	
	$ND_4(G)$	0.571	
	$ND_5(G)$	0.662	
EV	$NM_{1}(G)$	0.858	$ND_1(G)$
	$NM_{2}(G)$	0.439	
	*NABC*(*G*)	0.857	
	$NF^{*}(G)$	0.851	
	$ND_1(G)$	0.859	
	$ND_2(G)$	0.404	
	$ND_3(G$	0.704	
	$ND_4(G$	0.517	
	$ND_5(G)$	0.556	
MP	$NM_{1}(G)$	0.040	No best predictore
	$NM_{2}(G)$	0.037	
	*NABC*(*G*)	0.029	
	$NF^{*}(G)$	0.026	
	$ND_1(G)$	0.041	
	$ND_2(G)$	0.035	
	$ND_3(G)$	0.019	
	$ND_4(G)$	0.026	
	$ND_5(G)$	0.037

**Table 7:** Outcomes of Logarithmic Regression Model.

Property	Predictors	$R^{2}$	Best Predictor
BP	$NM_{1}(G)$	0.598	No best predictor
	$NM_{2}(G)$	0.467	
	*NABC*(*G*)	0.557	
	$NF^{*}(G)$	0.546	
	$ND_1(G)$	0.601	
	$ND_2(G)$	0.425	
	$ND_3(G)$	0.477	
	$ND_4(G)$	0.289	
	$ND_5(G)$	0.515	
MV	$NM_{1}(G)$	0.313	No best predictor
	$NM_{2}(G)$	0.123	
	*NABC*(*G*)	0.361	
	$NF^{*}(G)$	0.354	
	$ND_1(G)$	0.314	
	$ND_2(G)$	0.093	
	$ND_3(G)$	0.352	
	$ND_4(G)$	0.027	
	$ND_5(G)$	0.173	
MR	$NM_{1}(G)$	0.773	$ND_1(G)$
	$NM_{2}(G)$	0.643	
	*NABC*(*G*)	0.721	
	$NF^{*}(G)$	0.717	
	$ND_1(G)$	0.774	
	$ND_2(G)$	0.594	
	$ND_3(G)$	0.640	
	$ND_4(G)$	0.436	
	$ND_5(G)$	0.702	
MW	$NM_1(G)$	0.449	No best predictor
	$NM_2(G)$	0.551	
	*NABC*(*G*)	0.343	
	$NF^{*}(G)$	0.350	
	$ND_1(G)$	0.446	
	$ND_2(G)$	0.539	
	$ND_3(G)$	0.267	
	$ND_4(G)$	0.491	
	$ND_5(G)$	0.550	
HAC	$NM_{1}(G)$	0.747	$ND_5(G)$
	$NM_{2}(G)$	0.805	
	*NABC*(*G*)	0.622	
	$NF^{*}(G)$	0.626	
	$ND_1(G)$	0.746	
	$ND_2(G)$	0.779	
	$ND_3(G)$	0.518	
	$ND_4(G)$	0.670	
	$ND_5(G)$	0.818	
CO	$NM_{1}(G)$	0.573	No best predictor
	$NM_{2}(G)$	0.565	
	*NABC*(*G*)	0.489	
	$NF^{*}(G)$	0.483	
	$ND_1(G)$	0.575	
	$ND_2(G)$	0.538	
	$ND_3(G)$	0.403	
	$ND_4(G)$	0.430	
	$ND_5(G)$	0.580	
EV	$NM_{1}(G)$	0.551	No best predictor
	$NM_{2}(G)$	0.428	
	*NABC*(*G*)	0.516	
	$NF^{*}(G)$	0.508	
	$ND_1(G)$	0.553	
	$ND_2(G)$	0.388	
	$ND_3(G)$	0.446	
	$ND_4(G)$	0.263	
	$ND_5(G)$	0.474	
MP	$NM_{1}(G)$	0.028	No best predictor
	$NM_{2}(G)$	0.032	
	*NABC*(*G*)	0.018	
	$NF^{*}(G)$	0.015	
	$ND_1(G)$	0.029	
	$ND_2(G)$	0.032	
	$ND_3(G)$	0.008	
	$ND_4(G)$	0.023	
	$ND_5(G)$	0.028	

## Statistical techniques and regression model

The regression models were carefully tested and evaluated using three different regression models to arrive at the best and reliable model to estimate the relationship between the physical and chemical properties and the corresponding topological indices.

1 $$\begin{aligned} P&= \alpha + \beta (T) \end{aligned}$$

2 $$\begin{aligned} P&= \alpha + \beta (T) + \gamma (T)^2 \end{aligned}$$

3 $$\begin{aligned} P&= \alpha + \beta (lnT) \end{aligned}$$

P is a physicochemical property in the suggested regression models. T is a topological index, and *α*, *β* and *γ* are constants of the model. In the linear regression framework, *α* is the intercept and *β* is the regression coefficient, and the model’s performance is checked using the F-statistic (F-value), correlation coefficient (R), p-value (p), coefficient of determination $(R^{2})$, and standardized residuals (S). For the quadratic regression model, *α* denotes the constant term, *β* represents the coefficient of the topological index. The *γ* represents the coefficient of the squared index. To check how good the models are and find any unusual results, we used standardized residuals. These are calculated by dividing the residuals by their standard deviation. The parameter N represents the number of observations used in the regression analysis. The conventional criteria were used to define statistical significance: a p-value that is less than or equal to 0.05 shows the relationship between the predictor and the response variable is statistically significant, whereas an F-value that is more than 2.5 indicates that the model has a meaningful advantage over a null model that does not have predictors. The direction and strength of the association are determined by the correlation coefficient R, where the value of 0.8 or less is a strong positive or negative correlation respectively and the value is above 0.8 is a strong relationship. Tables 8, 9 and 10 provide the regression equations and their statistical parameters of the topological indices.

The results demonstrate that, within the linear regression framework, The selected topological indices were employed to predict the physicochemical properties of antidepressant drugs, as presented in Table 5. According to Table 6, the logarithmic regression model identifies $ND_1(G)$ and $ND_5(G)$ as significant predictors. Furthermore, the quadratic regression analysis, shown in Table 7, reveals that the indices $ND_1(G)$ and $ND_5(G)$ provide the most reliable estimations.

**Table 8:** Best predictors of linear regression models statistically calculated.

Property	Regression Equation	N	*α*	*β*	p	F	R	$R^{2}$	S_res_
BP	2.579($ND_1(G)$)+105.111	25	105.111	2.579	0.001	78.501	0.879	0.773	45.537
MR	0.539($ND_1(G)$) +17.589	25	17.589	0.539	0.001	149.980	0.931	0.867	6.887
MW	5.623($ND_5(G)$)+9.439	25	9.439	5.623	0.001	35.885	0.781	0.609	56.811
HAC	0.382($ND_5(G)$)+2.623	25	2.623	0.382	0.001	141.072	0.927	0.860	1.949
CO	4.287($ND_1(G)$)−197.708	25	−197.708	4.287	0.001	57.697	0.846	0.715	88.307
EV	0.313($ND_1(G)$)+29.804	25	29.804	0.313	0.001	61.113	0.852	0.727	6.263

**Table 9:** Best predictors of quadratic regression models statistically calculated.

Property	Regression Equation	N	*α*	*β*	p	F	R	$R^{2}$	S_res_	*γ*
BP	0.018($ND_1(G)$)$^{2}$−1.864($ND_1(G$)+367.084	25	367.084	−1.864	0.001	86.291	0.942	0.887	32.889	0.018
MR	0.001($ND_1(G)$)$^{2}$+0.271($ND_1(G)$)+33.369	25	33.369	0.271	0.001	78.872	0.937	0.878	6.756	0.001
CO	0.022($ND_1(G)$)$^{2}$−1.273($ND_1(G)$)+130.115	25	130.115	−1.273	0.001	37.769	0.880	0.774	80.323	0.022
EV	0.002($ND_1(G)$)$^{2}$−0.287($ND_1(G)$)+65.175	25	65.175	−0.287	0.001	66.793	0.927	0.859	4.605	0.002

**Table 10:** Best predictors of logarithm regression models statistically calculated.

Property	Regression Equation	N	*α*	*β*	p	F	R	$R^{2}$	S_res_
MR	−178.770+55.169($lnND_1(G)$)	25	−178.770	55.169	0.001	78.786	0.880	0.774	8.978
HAC	−44.647+17.125($lnND_5(G)$)	25	−44.647	17.125	0.001	103.496	0.905	0.818	2.219

**Fig. 2:**
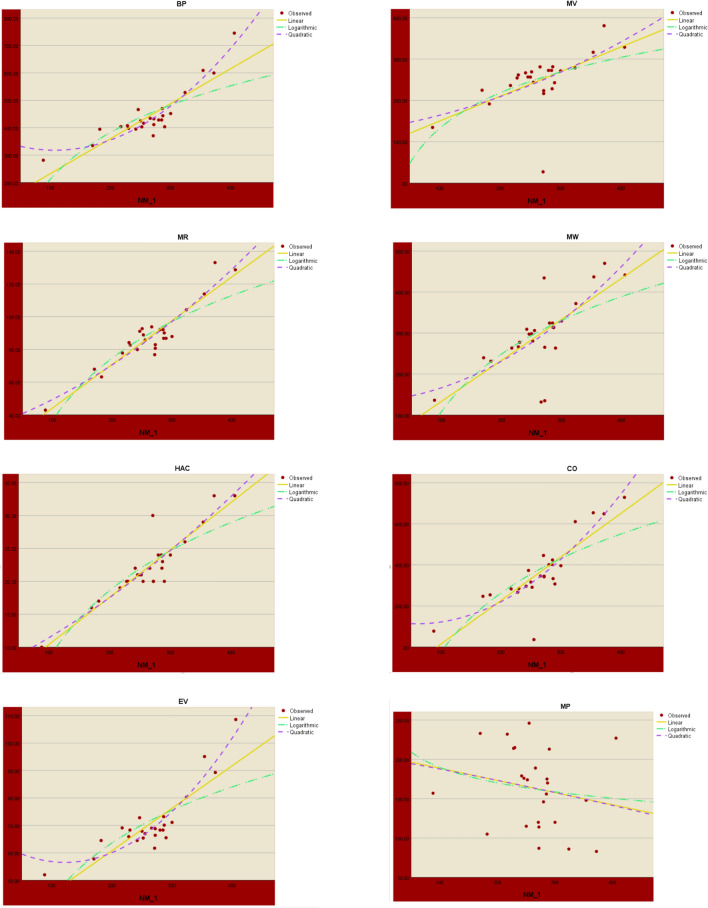
Regression analysis conducted on the $NM_1(G)$ index.

**Fig. 3:**
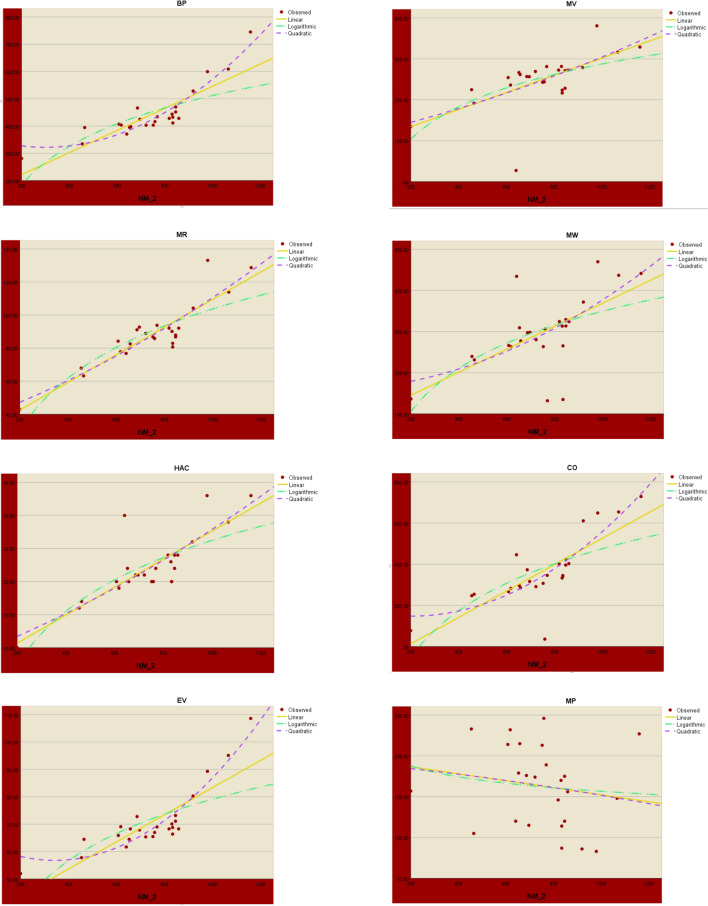
Regression analysis conducted on the $NM_2(G)$ index.

**Fig. 4:**
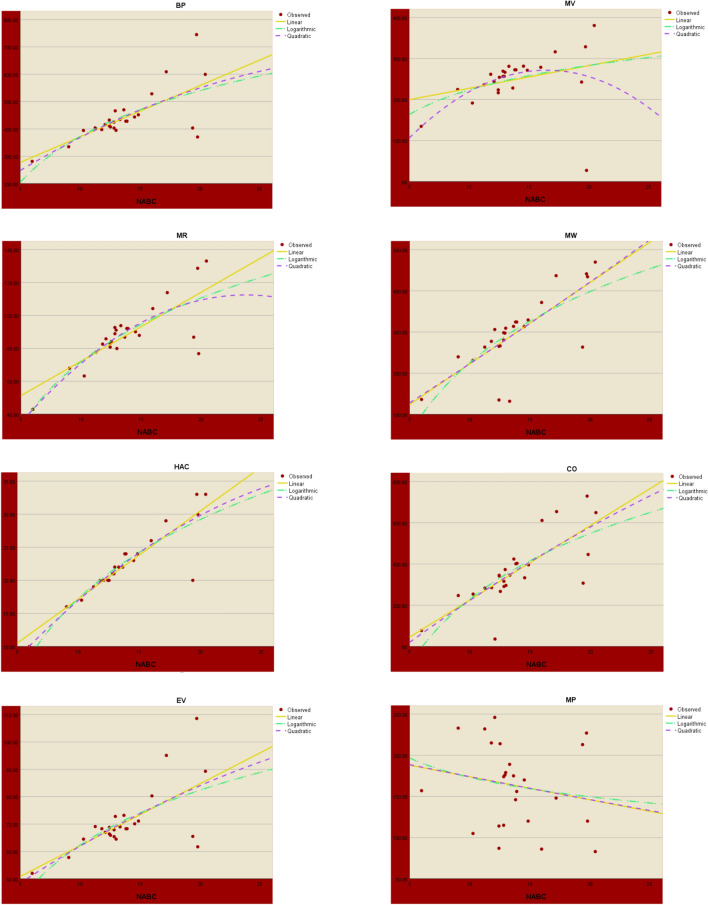
Regression analysis conducted on the *NABC*(*G*) index.

**Fig. 5:**
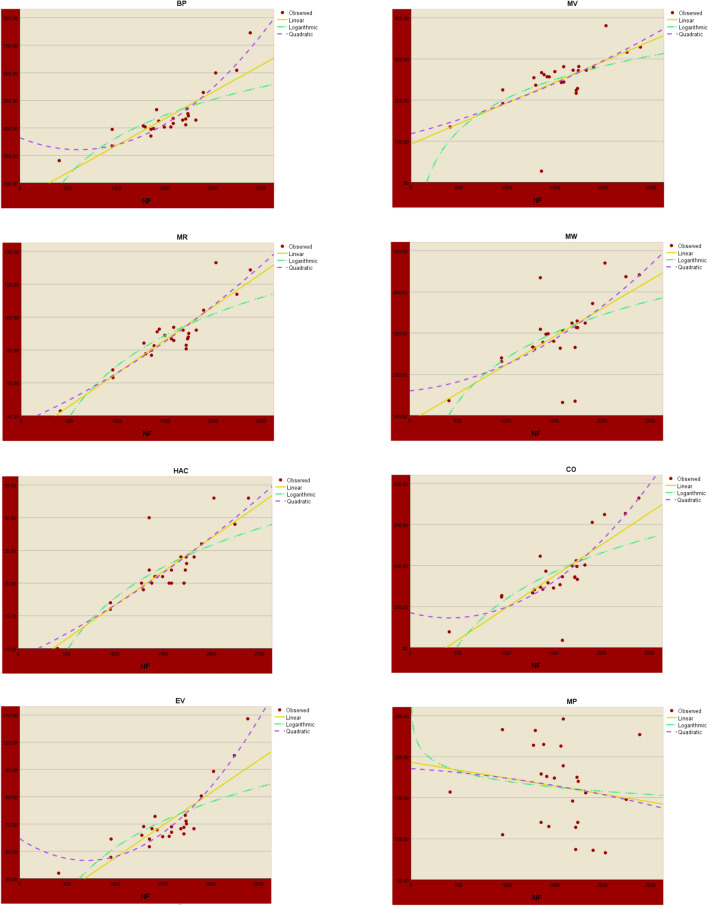
Regression analysis conducted on the $NF^*(G)$ index.

**Fig. 6:**
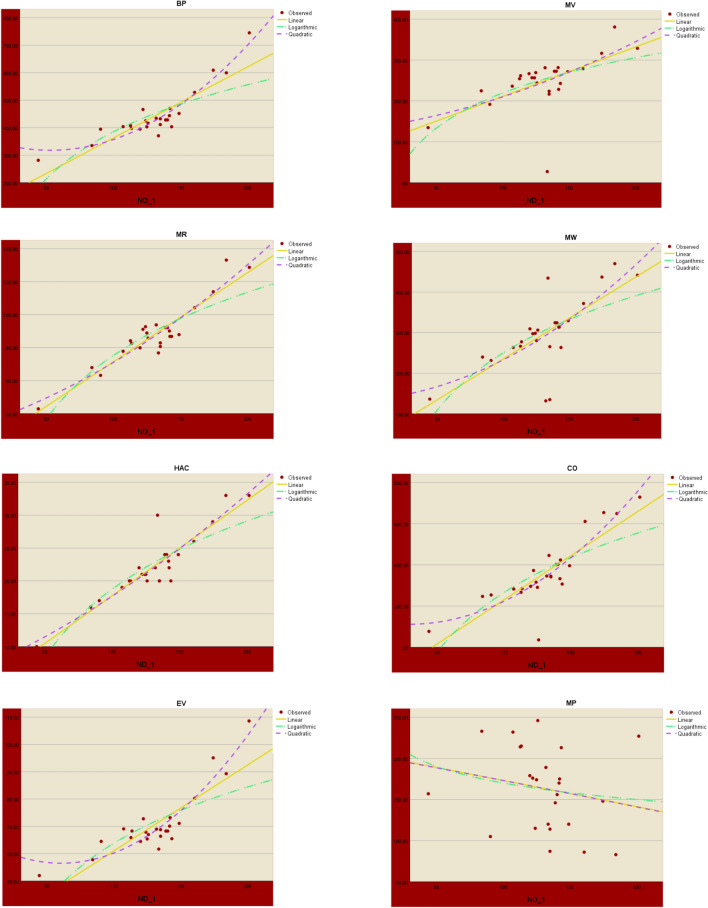
Regression analysis conducted on the $ND_1(G)$ index.

**Fig. 7:**
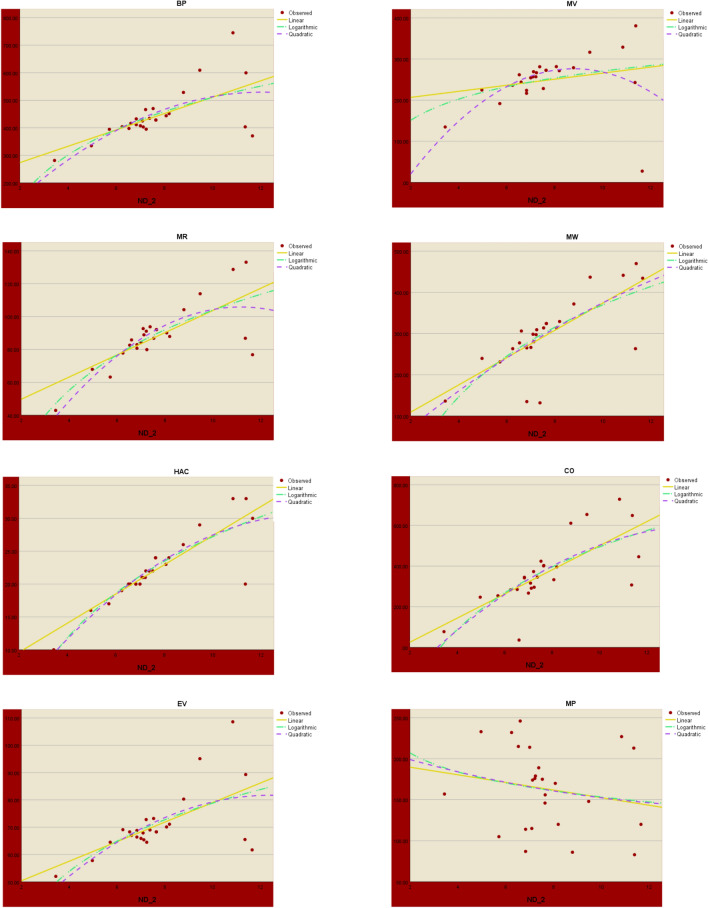
Regression analysis conducted on the $ND_2(G)$ index.

**Fig. 8:**
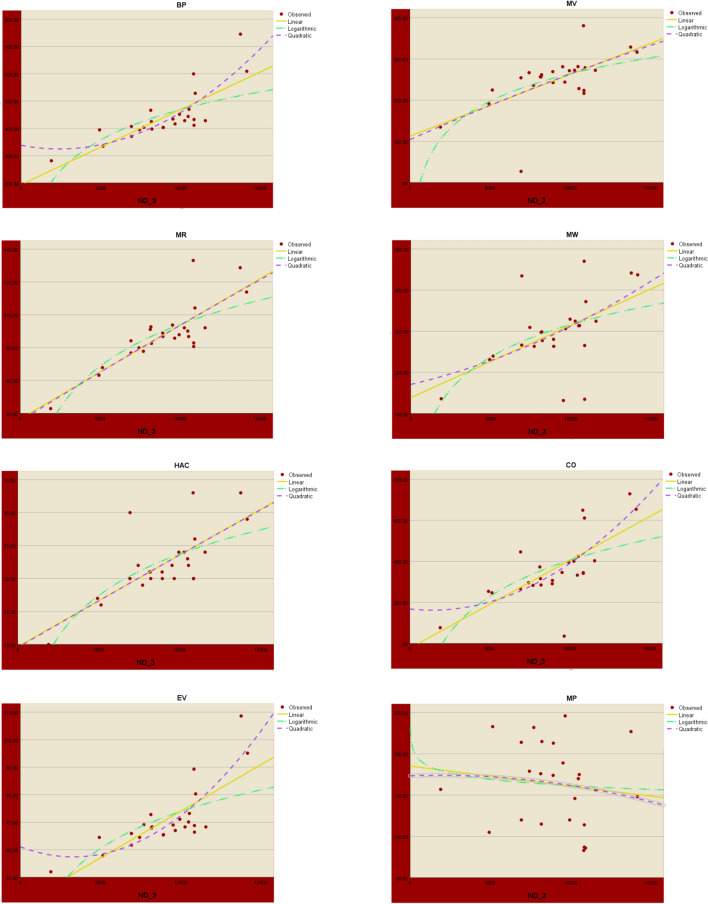
Regression analysis conducted on the $ND_3(G)$ index.

**Fig. 9:**
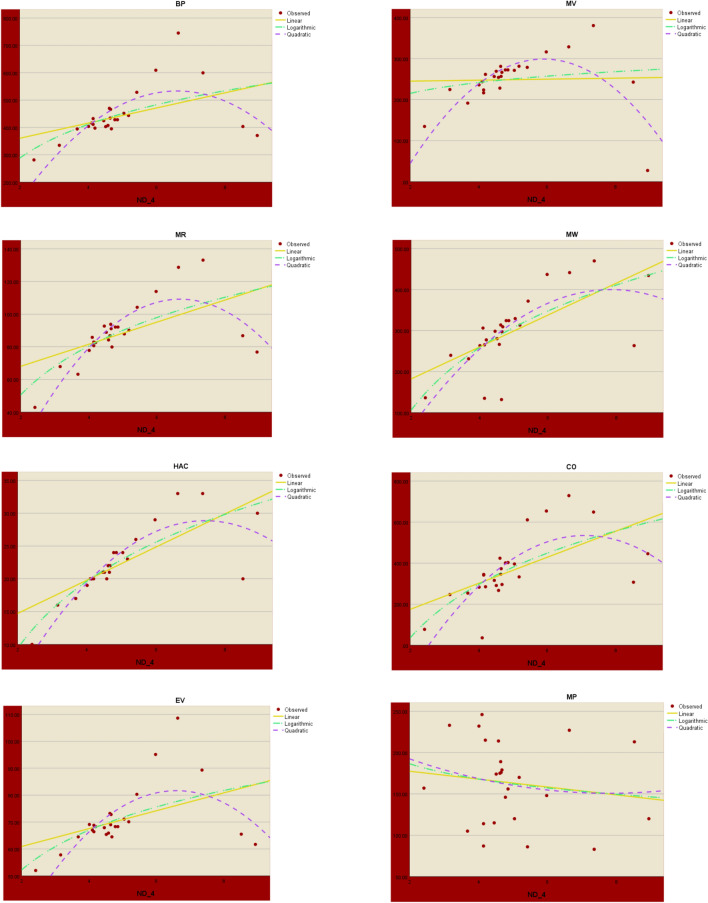
Regression analysis conducted on the $ND_4(G)$ index.

**Fig. 10:**
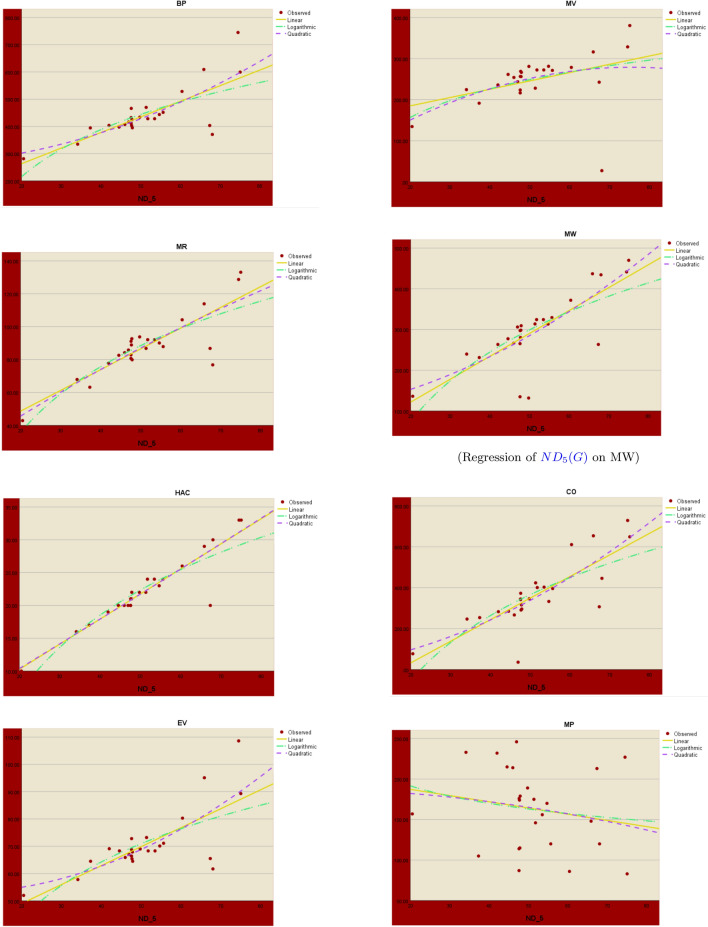
Regression analysis conducted on the $ND_5(G)$ index.

The regression analyses of neighborhood degree based topological indices are shown graphically in Figs. 2, 3, 4, 5, 6, 7, 8, 9, 10 for linear, quadratic and logarithmic models respectively. That is, the regression analysis was performed on ($NM_1(G)$), as shown in Fig. 2, ($NM_2(G)$) in Fig. 3, and (*NABC*(*G*)) in Fig. 4. Similarly, the regression analyses for ($NF^{*}(G)$) and ($ND_1(G)$ are shown in Figs. 5 and 6 respectively, and Figs. 7 and 8 show the regression analyses for ($ND_2(G)$) and ($ND_3(G)$), respectively. Lastly, the regression analyses of ($ND_4(G)$) and ($ND_5(G)$), respectively, are shown in Figs. 9 and 10. The information displayed in these graphical representations shows the general performance of the three regression models and shows how well each model describes the relationship between the response variables and the neighborhood indices to which they are related. The figures illustrate how the observed trends and spread of data points can be used to assess the predictive ability of the model and to see how well the model reflects the structural and physicochemical properties of the compounds being examined.

After analyzing the indices in the quadratic regression model in Table 6, the results show that defining $ND_1$ makes it possible to create the following models to predict BP, MR, CO, and EV:

4 $$\begin{aligned} {BP}&= {0.018\left( ND_1(G)\right) ^2 - 1.864\,ND_1(G) + 367.084}, \end{aligned}$$

5 $$\begin{aligned} {MR}&= {0.001\left( ND_1(G)\right) ^2 + 0.271\,ND_1(G) + 33.369},\end{aligned}$$

6 $$\begin{aligned} {CO}&= {0.022\left( ND_1(G)\right) ^2 - 1.273\,ND_1(G) + 130.115},\end{aligned}$$

7 $$\begin{aligned} {EV}&= {0.002\left( ND_1(G)\right) ^2 - 0.287\,ND_1(G) + 65.175}. \end{aligned}$$

### Comparative analysis of regression approaches

A methodical analysis was carried out in order to determine the most appropriate regression model to use to predict the relationship between topological indices and each of the eight physicochemical properties of the antidepressant compounds. Linear, quadratic and logarithmic regression models were compared using the coefficient of determination $R^2$. Table 11 highlights the regression model that produced the best predictive accuracy of each property. The results of the analysis validate the significance of topological indices as strong and understandable molecular descriptors in QSAR/QSPR analysis, which shows that well-chosen descriptors can provide predictable property information without the need to employ overly complicated models^20^.

**Table 11:** Optimal Regression Model for Each Physicochemical Property.

Property	Best Model	Best Predictor	$\mathbf {R^2}$	Improvement over Linear Model
Boiling Point (BP)	Quadratic	$ND_1(G)$	0.887	14.7% higher
Molar Volume (MV)	Quadratic	$ND_4(G)$	0.586	2.3% higher
Molar Refractivity (MR)	Quadratic	$ND_1(G)$	0.878	151.6% higher
Molecular Weight (MW)	Logarithmic	$ND_1(G)$	0.774	426.5% higher
Heavy Atom Count (HAC)	Linear/Quadratic	$ND_5(G)$	0.860	0% (tie)
Complexity (CO)	Quadratic	$ND_1(G)$	0.774	8.3% higher
Enthalpy of Vaporization (EV)	Quadratic	$ND_1(G)$	0.859	18.2% higher
Melting Point (MP)	Linear	$ND_1(G)$	0.041	Comparable to quadratic

With the properties of the **Complexity (CO)** and **Enthalpy of Vaporization (EV)**, it was observed that there was no model or predictor that exhibited a high predictive relationship of the properties in all indices (the values of the ($R^2$ were generally low), which implies that these properties might be affected by some factors beyond those that had been captured by the topological descriptors adopted.

The analysis has shown a number of significant tendencies:

- The quadratic regression model showed better performance on five of the eight key properties that were analysed (BP, MV, MR, MW and HAC). This result shows that the correlations between the molecular descriptors and these physicochemical properties are mostly curvilinear, but not necessarily linear.
- On the contrary a linear model fitted best in predicting melting point. This implies that the energy related to crystalline lattice formation can be more directly proportional to the structural characteristics taken by the index of the $ND_4(G)$.
- Logarithmic regression showed minimal advantage in this context, achieving optimal performance only in isolated instances. This implies that exponential scaling relationships between the descriptors and properties are less common within this specific dataset of antidepressant compounds.
- The predictive power of the models varied significantly across properties, with excellent results for MW, MP, and HAC, but limited utility for CO and EV, highlighting the selectivity of topological indices.

### Ranking of topological indices by predictive power

In order to evaluate the overall effectiveness of each of the topological indices under study, each index was evaluated with regards to its mean value of the mean of $R^2$ across all the physicochemical properties and regression models. This overall analysis enabled the nine indices to be grouped into three levels in a classification system as shown in Table 12. The measures the effectiveness of the known topological indices in representing molecular structure and branch structures.It points out the necessity of properly interpreting the indices to make the accurate predictions of chemical properties^21^.

**Table 12:** Leveled Separating Topological Indices by Predictive Power.

Tier	Indices	Average $R^2$	Primary Predictive Strengths
Tier 1 (Excellent Predictors)	$ND_1(G)$, $NM_1(G)$, *NABC(G)*	0.75–0.80	Boiling point, molar refractivity, enthalpy of vaporization, heavy atom count
Tier 2 (Good Predictors)	$ND_4(G)$, $ND_5(G)$, $\ NF^*(G)$	0.58–0.68	Heavy atom count, molecular weight, complexity
Tier 3 (Moderate Predictors)	$ND_2(G)$, $NM_2(G)$, $ND_3(G)$	0.45–0.55	Limited applicability; moderate correlations with selected properties

Several critical observations emerge from this ranking:

- There exists a definite tendency that indicates that indices which used the degree of a vertex, i.e. (*ND* series, had a higher predictive accuracy than indices based on adjacency or other structural measures. This high-performance can probably be attributed to the capability of these to encode more efficiently basic information on molecular connectivity and branching pattern characteristic of antidepressant scaffolds studied.
- The $ND_4(G)$ was the **most reliable and versatile** among all the indices. It had large values of $R^2$ to predict four different properties which highlights its wide applicability that makes it useful in the quantitative structure-property relationship (QSPR) modeling of this group of compounds.
- The index *ND3* was determined to possess the **lowest overall predictive strength** throughout the research. This finding suggests that the molecular specificity of the information coded in the form of the specific molecule, *ND3*, does not relate to the physicochemical properties of antidepressant molecules as directly as may have been expected, and as therefore is not useful as a general description of this chemical domain.

### Practical application guide for rational drug design

The insights obtained from the comparative analysis can serve as a practical guide for medicinal chemists in future studies. The findings have been summarized into a practicable selection guide based on the desired modification of specific physicochemical properties during the optimization of lead in Table 13 with the aid of modern computational and experimental methods such as QSAR, molecular modeling, and artificial intelligence. It also speaks of more effective therapeutic development using advanced strategies; like PROTACs, and structure-based methods, which develop more efficiently and creatively^22^using computational chemistry to simplify drug discovery, with particular interest in such techniques as molecular modeling, docking, and QSAR to predict drug-target interactions. It also directs medicinal chemists on how to use such tools to design and optimize useful therapeutic molecules^23^. This guide is expected to be used as a point of departure into computational screening. As an example, in cases where the design objective is to enhance the solubility of a compound a property that is highly dependent on hydrogen bonding concentrating computational resources on the index of the *NABC(G)* in a quadratic modeling model is suggested. Equally, the $blue{ND_5(G)}$ descriptor would come in handy in a project whose goal is to closely regulate the size of a molecule in the course of synthesis. The suggested secondary indices present complementary information which may give a more global understanding of the structure-property landscape.

**Table 13:** Topological Index Guide to Molecular Design Targeting.

Molecular Design Objective	Primary Recommended Index	Secondary Recommended Index	Recommended Model
Modulate Aqueous Solubility	*NABC(G)* (informs H-bonding capacity)	$ND_5(G)$ (informs heavy atom count)	Quadratic
Optimize Oral Bioavailability	$ND_1(G)$ (informs boiling point & enthalpy)	*NABC(G)* (informs H-bonding capacity)	Quadratic
Adjust Molecular Size/Weight	$ND_1(G)$ (predicts molecular weight)	$ND_5(G)$ (predicts molecular weight)	Logarithmic/Quadratic
Estimate Metabolic Stability	$NM_1(G)$ (informs molecular shape & complexity)	*NABC(G)* (informs molecular irregularity)	Quadratic
Predict Boiling Point Behavior	$ND_1(G)$ (best BP predictor)	*NABC(G)* (strong BP predictor)	Quadratic
Predict Enthalpy of Vaporization	$ND_1(G)$ (best EV predictor)	*NABC(G)* (strong EV predictor)	Quadratic

## Leave-one-out cross-validation

A model evaluation method that is applied to see how well a regression model predicts the outcome of a model that it has never been exposed to when it was being trained, as opposed to how well the model fits the data it was trained on. Cross validation is also required in the case of QSPR modeling model, which uses a topological index, which is a numerical description of the structure of a drug molecule, to predict physicochemical or biological properties, since the data sets used are typically limited in size and can easily overfit the model. The most popular method, K-Fold cross validation, is a way of splitting the data into multiple folds or subsets of equal size. The model is trained on the remaining 7 folds and tested on the 8th fold, then the process is repeated until the 7th fold is used for testing once. The errors (e.g., MSE or RMSE) of each fold are averaged and the average error serves as a measure of the model’s predictive reliability, and the cross-validated Q² value shows how much variation of the property that can be attributed to the structural descriptor and not coincidence.

One of the special cases of K-Fold cross validation is Leave-One-Out Cross Validation, in which the number of folds is equal to the number of data points in the dataset. For each iteration, one drug molecule (one data point) is omitted from the data set and used as a test molecule, while the model is trained with all the other molecules; this process is repeated until all the drug molecules have been omitted and tested exactly once. However, each test set has only one observation and hence LOOCV yields an almost unbiased estimate of the performance of the model and is especially suitable in the QSPR studies where the data sets consist of drug compounds and all the data points have valuable information. The price is computational effort (because the model needs to be re-evaluated as many times as there are data points), but the effort is small and valuable in the context of small to moderate sized datasets common in topological index–based QSPR research for the precision in error estimation achieved (LOOCV-Q², average LOOCV MSE, etc.).

The importance of the use of cross validation and LOOCV in QSPR models constructed on topological indices is that they can provide a measure of confidence that the model is applicable to actual prediction of a new untested drug molecule. During a drug discovery process, the synthesis and experimental testing of all possible candidates is costly and time consuming, so if a validated QSPR model exists, it can be used to virtually test large libraries of molecules, and narrow down to a handful of the most promising candidates for experimental testing. For a structural descriptor to be considered robust and reliable, it should consistently produce high Q² values and low cross-validated errors in both the K-Fold and LOOCV procedures, and thus it is unlikely that the correlation with the target drug property, such as toxicity, solubility or bioactivity, is a statistical fluke due to the nature of the training data. This demonstrated reliability is the reason why a topological-index-based QSPR model is used with confidence in early-stage pharmaceutical research, saving costs and time on the traditional trial and error method of drug development are shown in Table 14.

**Table 14:** Leave one out cross validation results.

Regression equations	$R^2$	$Q^2$	*R*	RMSE	MSE
2.579($ND_1(G)$)+105.111	0.773	0.7734	0.879	43.6775	1907.721
0.539($ND_5(G)$) +17.589	0.867	0.867	0.931	6.6059	43.6375
5.623($ND_5(G)$)+9.439	0.609	0.7735	0.781	34.7216	1205.5899
0.382($ND_5(G)$)+2.623	0.860	0.8598	0.927	1.8692	3.4941
4.287($ND_1(G)$)−197.708	0.715	0.715	0.846	84.7012	7174.2859
0.313($ND_1(G)$)+29.804	0.727	0.7266	0.852	6.0071	36.0848
0.018($ND_1(G)$)$^{2}$−1.864($ND_1(G)$)+367.084	0.887	0.7109	0.942	49.3352	2433.9583
0.001($ND_1(G)$)$^{2}$+0.271($ND_1(G)$)+33.369	0.878	0.8006	0.937	8.0904	65.4545
0.022($ND_1(G)$)$^{2}$−1.273($ND_1(G)$)+130.115	0.774	0.6845	0.880	89.1132	7941.1677
55.169($lnND_1(G)$)−178.770	0.774	0.772	0.880	8.6116	74.1597
17.125($lnND_1(G)$)−44.647	0.818	0.8182	0.906	2.1287	4.5313

## Conclusion

A detailed QSPR study of twenty-five antidepressant drugs using nine neighborhood degree-based topological indices obtained from chemical graph theory was presented in this study. Essential structural properties of the molecular graphs were captured by the computed descriptors and linear, quadratic, and logarithmic regression models were built for eight important physicochemical properties.

The quadratic regression model was always the best predictor for most of the physicochemical properties, suggesting that the relationship between molecular topology and physicochemical behavior is better described by a nonlinear model. Specifically, the neighborhood descriptors $ND_1(G)$ and $ND_1(G)$ showed the best predictive ability for various properties, and the most predictive ability in terms of $R^{2}$ value was boiling point (0.887). The prediction of melting point and some other properties was still relatively poor, but the overall results showed that the neighborhood-based topological indices are structural information for reliable prediction of the QSPRs.

The suggested models provide a fast and cost-effective computational tool for estimating physicochemical properties from molecular graph directly without performing extensive laboratory experiments. Thus, these descriptors could be used as useful tools for virtual screening, lead optimization, and rational design of new antidepressant drugs. These graph-theoretical descriptors can be further enhanced by combining with other molecular descriptors, machine learning methods and expanded molecular database to build more powerful and generalizable QSPR model for future studies.

The results presented in this research could be further extended by incorporating larger and more divers set of antidepressant compounds including newly developed drug molecules which can further enhance the robustness and generalizability of the proposed QSPR models. Adopting neighborhood degree-based topological indices alongside sophisticated machine learning and artificial intelligence models, including random forests, support vector machines, and deep learning frameworks, could potentially further boost prediction accuracy. Furthermore, the use of graph-theoretical descriptors together with quantum chemical, 3D and electronic molecular descriptors may offer a more complete picture of the molecular structure and enhance the prediction of more complex, physicochemical molecular properties, such as melting point and molecular complexity. These could help to make virtual screening more reliable, rational drug design, and the discovery of safer and more effective antidepressant agents faster.

## Data Availability

The data that support the findings of this study are available from the corresponding author upon reasonable request.
